# Natural radioactivity and associated hazard indices from a gas field (Fenchuganj, Bangladesh): Assessment and management guidelines

**DOI:** 10.1371/journal.pone.0358600

**Published:** 2026-09-17

**Authors:** Mahbuba Begum, Shahadat Hossain, Md. Bazlar Rashid, Md. Ahosan Habib, Rahat Khan, M.M. Mahfuz Siraz, Selina Yeasmin, S.M. Mostafa Al Mamun, Syed Mohammod Hossain

**Affiliations:** 1 Health Physics Division, Atomic Energy Centre Dhaka, Bangladesh Atomic Energy Commission, Shahbag, Dhaka, Bangladesh; 2 Institute of Materials Structure Science, High Energy Accelerator Research Organization (KEK), Tsukuba, Ibaraki, Japan; 3 Geological Survey of Bangladesh, Segunbagicha, Dhaka, Bangladesh; 4 Institute of Nuclear Science & Technology, Bangladesh Atomic Energy Commission, Savar, Dhaka, Bangladesh; 5 Department of Electrical and Electronic Engineering, University of Dhaka, Dhaka, Bangladesh; Cameroon National Radiation Protection Agency, CAMEROON

## Abstract

It is crucial to carry out thorough research and risk assessments in the oil and gas production and processing sectors because of the possible health risks associated with the redistribution of naturally occurring radioactive materials (NORMs). Radioactivity levels in surface soil and core samples taken from Bangladesh’s Fenchuganj gas field were assessed in this comprehensive investigation. The concentrations of radionuclides were determined using high-purity germanium (HPGe) gamma-ray spectrometry. Surface soil samples have activity concentrations of ^226^Ra, ^232^Th, and ^40^K ranging from 16–32 Bq kg^-1^, 18–78 Bq kg^-1^, and 144–516 Bq kg^-1^, respectively, while core samples have activity concentrations ranging from 31–62 Bq kg^-1^, 52–93 Bq kg^-1^, and 1043–2219 Bq kg^-1^, respectively. The core samples’ activity concentrations exceeded the average soil value for the world. These high levels are caused by a variety of factors, including fluid dynamics, geological structures, hydrocarbon reserves, geological composition, and human activities associated with gas production. According to the assessment of radiological risk indices, some of them are higher than the advised thresholds. The distribution features of the radioactive concentrations were investigated using statistical techniques, such as descriptive statistics, principal component analysis, and hierarchical cluster analysis. To safeguard the environment and occupational exposure, appropriate management measures, including regular radiological monitoring, dust control, safe handling of core materials, and worker radiation safety practices, are recommended. Overall, the study emphasizes how crucial it is to understand and monitor the radiological hazards due to gas extraction operations in Bangladesh in order to protect both employees and the local population.

## Introduction

Environmental pollution has received more attention in the last few decades due to growing population demands and the growth of various industrial processes, including the extraction of minerals and the production of gas and oil. Prior research works have shown that the industrial wastes from the coal, gas and oil exploration industries have increased the amount of radiological and chemical harmful materials in the surrounding environment, which can eventually endanger the health of the inhabitants and workers [1–3]. Now, produced water is recognized as the main waste stream in gas and oil producing industries. It includes both organic and inorganic compounds, as well as dissolved and dispersed oils, scale products, waxes, radionuclides, heavy metal & metalloids, dissolved gases, salts, formation solids, microbes, treatment chemicals, and dissolved oxygen [3–8]. The physical and chemical characteristics of produced water can differ significantly based on geological age, depth, hydrocarbon-bearing formation geochemistry, and the supplementary production chemicals and chemical composition in the gas and oil phase reservoir [9]. Drilling waste generated by the hydrocarbon exploration industries is the second largest amount of waste [10]. It is considered that the hydrocarbon producing field evaporation pond is the primary source of NORMs and hazardous element wastes since all drilling waste, including mud, brine and produced water, is dumped into it [3]. Environmental contamination of the local pedosphere occurs when this contaminated water overflows, usually during the rainy season, and spreads to surrounding ponds and soil.

The wastes generated by the gas and oil exploration industries are putting a supplementary load of NORMs in the ambient environment. Since we know that the earth’s crust contains natural radionuclides that have existed from the beginning of time. All living things are exposed to natural radiation primarily because of the radioactivity concentration of primordial natural radionuclides, which have longer half-lives, such as ^232^Th, ^40^K, ^238^U, and their decay products [11]. According to Begum et al., the radioactivity concentration levels of NORMs can exceed the surface background by the hydrocarbon production process, where scale and sludge either accumulate with the silicate, carbonate, or sulphate of alkaline earth metals and precipitate in storage tanks, pipelines, or else by flowing through produced water [12]. The concentration of subsurface radionuclides and the chemical makeup of the formation fluid determine the radioactivity of the waste stream from gas and oil production; once more, the extraction, treatment, and production duration alter the temperature and pressure, which in turn alters the concentration of NORMs [13]. The quantity of ^226^Ra activity in hydrocarbon abstraction wastes, the main radioisotope of concern, ranges widely from undetectable to 1000 kBq kg^-1^ and in another scientific study of identical industrial wastes, ^226^Ra activity concentration reached up to 15 MBq kg^-1^ [4,12,14]. Depending on a number of factors, such as geological formation, operational condition, etc., the gas and oil production field can vary significantly from one another [4,5,12,15–17]. Hence, it is imperative to monitor the radioactivity concentration of hydrocarbon production fields in the modern world.

In the oil and gas industry, managing and disposing of scales and sludge during maintenance and decommissioning operations is the major source of radiation protection concerns. The necessity for strict radiation protection measures is highlighted by the substantial danger that these procedures offer to personnel owing to dust inhalation and exposure to external gamma radiation. Similar precautions could also be necessary for workers near pipelines and vessels that have a lot of scaling because of the high radiation levels. In addition, the majority of the focus on technologically enhanced NORM (TENORM)-related health concerns and hazards in oil and gas production centers on the generation and release of radon (^222^Rn). The degree of this risk relies on several variables, such as the rate at which ^222^Rn is released into the environment and the radiation emitted from the resulting waste matrix that contains TENORM; thus, careful consideration and management are required. The extraction of gas and oil produces TENORMs, which are wastes that are often released into the environment over long periods of time. These dumps for waste are often found within a few kilometers of both vast agricultural plains and highly inhabited areas. Natural radionuclides have the potential to gradually seep into adjacent bodies of water, such as lakes and rivers that are used for agricultural or public access. Significant volumes of produced water containing radioactive materials may also be directly released, posing a danger of contamination of surface and subterranean water sources. Crops, vegetables, and animals can all be negatively impacted by agricultural operations that increase the spread of contaminants into the land environment through a variety of pathways. This raises concerns about the possibility of radiation exposure in humans from both natural and man-made sources. The radioactive risks posed by the TENORM, including wastes from oil and gas extraction, must thus be carefully considered.

However, it is crucial to conduct radioactivity studies on oil and gas field core samples for a few reasons [18]. In order to determine if radioactive elements may be present in oil and gas sources, radioactivity analysis is first helpful. To understand the possible health and environmental risks associated with the extraction and use of oil and gas reserves, it is essential to accurately determine the concentrations and dispersion patterns of radioactive elements. Additionally, analyzing the radioactivity of core samples might aid in lowering the risks and uncertainties related to reservoir development. Improved reservoir modelling and more precise recovery factor forecasts result from a deeper comprehension of the reservoir’s properties and behavior. To the best of our knowledge, the crucial significance of assessing radioactivity in core samples inside gas fields has received little attention globally [3]. By performing a comprehensive radioactivity investigation on core soil samples taken from the Fenchuganj gas field (FGF), the current study seeks to bridge this gap.

Natural gas is by far the most abundant natural resource of Bangladesh. Out of the 29 gas fields found in Bangladesh so far, gas was being extracted from 21 fields [19]. Baseline data on radiation levels in Bangladesh’s gas fields and core samples taken from these fields are notably lacking [3,20,21]. Thus, this study offers the investigation of radioactivity levels and possible health risks associated with radiation exposure in surface soil and gas well core samples taken from Bangladesh’s FGF, which yields 28,13,944 L of produced water yearly [19]. Concurrently, this study aims to provide a comprehensive knowledge of the radiological effects from FGF and offers management guidelines to mitigate the consequences.

## Materials and methods

### Study area

**Fig 1:**
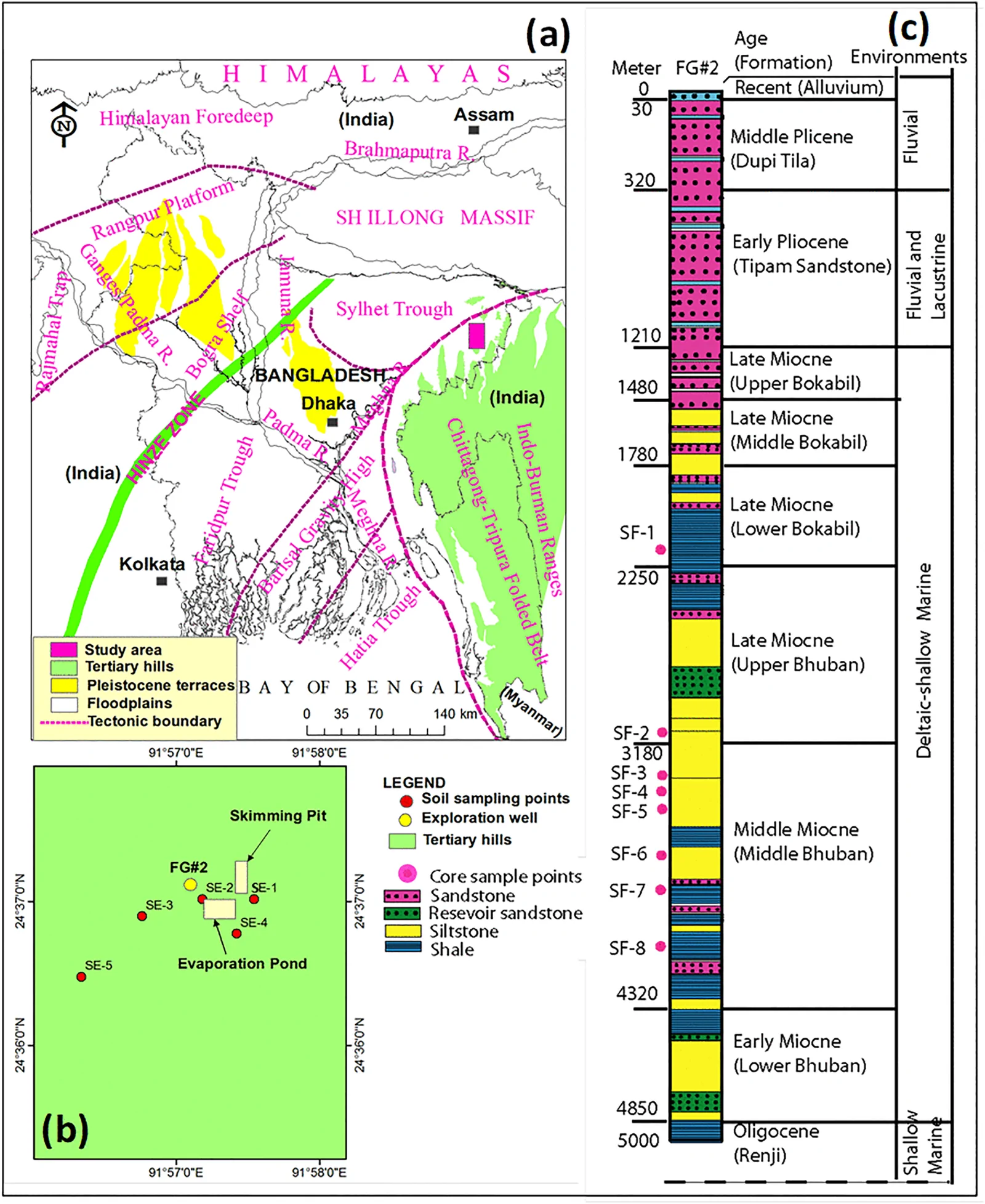
(a) Tectonic map of the Bengal Basin (data available from the U. S. Geological Survey & prepared by using ArcMap 10.2 and Adobe Illustrator CS and modified from Sikder and Alam [22], Uddin and Lundberg [23]); (b) study location; (c) stratigraphy, lithology and collected sample depths in the Fenchuganj gas field (well#2, compiled from BAPEX [24]).

The Fenchuganj area lies within the Chattogram-Tripura Fold Belt of the SE Bengal Basin (Bangladesh) and immediately west of the Indo-Myanmar deformation front (Fig 1). It is situated in the Sylhet trough/depression, which has been formed due to continuous uplift of the Shillong Massif with simultaneous subsidence [22]. This trough is situated in the south of the Shillong Massif and corresponds to the vast lowlands of the Surma valley. On the western edge of the Tripura high and in the south-central region of the Surma Basin are the FGF. It is a surface anticline, and the area is occupied by low hillocks and vegetation. The sediments of the Fenchuganj structure mainly consist of sand, silt and clay, in varying proportions of Oligocene to Recent age, and have a mineralogical composition of quartz, feldspars, illite and kaolinite [25]. It was evident from weathering profiles that silicate weathering predominated. “Transported soils” are created by transported alluvial deposits, which are primarily from the Holocene era (Recent), whereas “Residual soils” are created by native sediments obtained from the weathering of earlier hills [26]. The river system drains from the Tibetan Plateau. It passes through a variety of rock types, such as felsic intrusives, Paleozoic–Mesozoic sandstones, shales, and limestones, as well as Precambrian metamorphics (high-grade schists, gneisses, quartzites, and metamorphosed limestones) [27].

### Sample collection and processing

For this study, samples have been collected from “Bangladesh Petroleum Exploration and Production Company Limited (BAPEX)” through the approval authority, “Bangladesh Oil, Gas & Mineral Corporation (Petrobangla)”. Four surface soil samples (varying in depth from 0 to 6 inches) and one sediment sample were collected from the FGF in order to track local environmental radioactivity. The locations of the sampling points are described in Table 1 and Fig 1. In addition, eight core samples (Table 2 and Fig 1) were taken from six different cores at different depths inside the FGF boreholes. The samples were arranged according to the core number and depths of each. The selection of thirteen (13) samples was based on their spatial distribution across the Fenchuganj gas field to capture regional heterogeneity. Due to the logistical and technical challenges associated with core drilling and radiological assessments at such depths, this number reflects a practical limitation. At each sampling location, samples of topsoil (depth from surface level: 0–6 inches) or sediment weighing between 1.0 and 1.5 kg were taken. After being given numerical tags to make identification easier, they were allowed to dry in an electric oven at 60 °C to have constant weight. An agate mortar was used to crush and combine the samples of gas well core. Initially, tougher materials were crushed using a specialized hand mortar made of hardened alloy steel. Each cylindrical plastic jar containing the dried powdered sample weighed around 200 g. The containers were labelled with the proper identifying numbers, sealed around the cap with sturdy vinyl tape, and stored for a minimum of four weeks. A balanced condition between ^226^Ra in the ^238^U series and ^228^Ra in the ^232^Th series, together with their corresponding progeny, is ensured by this meticulous process. For the accurate measurement of radium and its progeny in the samples, this equilibrium condition is essential [28–31].

**Table 1:** Description of sampling points.

Sample ID	GPS of the location		Sample location description	Sample type
	Latitude (°)	Longitude (°)		
SE-1	24.617	91.959	Skimming pit zone	Surface soil
SE-2	24.617	91.953	Bottom of the evaporation pond	Sediment
SE-3	24.615	91.946	North-western side of evaporation pond	Surface soil
SE-4	24.613	91.957	South-eastern side of evaporation pond	Surface soil
SE-5	24.608	91.939	Outside of gas field and plant area	Surface soil

**Table 2:** Description of gas well core samples.

Well No.	Core No.	Core sample ID	Approximate depth from top surface (m)
2	4	SF-1	~ 2190
	7	SF-2	~ 3140
	8	SF-3	~ 3260
	8	SF-4	~ 3265
	8	SF-5	~ 3270
	10	SF-6	~ 3625
	11	SF-7	~ 3770
	12	SF-8	~ 4090

### Measurement procedures and data analysis

To assess the samples’ natural radioactivity, a p-type co-axial high purity Germanium (HPGe) detector (CANBERRA: Model GC-4019; serial no. 07089419) was used. The HPGe detector’s efficiency was 40%. The detector had an effective volume of 93 cm³ and showed 1.8 keV energy resolution for the ^60^Co γ-ray line’s 1332 keV energy peak. Software called Genie-2000 (Canberra) and MAESTRO-32 (ORTEC) were used to analyze the sample spectra. The “True Cascade Summing Correction" (CSC) function, self-attenuation via the LabSOCS (Lab System for On-site Counting System), and algorithms to account for radioactive decay are all included in Genie-2000. The detector’s effectiveness for various natural radionuclides was assessed using standard solid samples of Al_2_O_3_ and uniformly mixed standard solutions of ^226^Ra. All samples were counted for 20,000 seconds, and the sample and standard geometries were kept at a constant level. Point sources of ^152^Eu, ^60^Co, and ^137^Cs were used to calibrate the energy on the HPGe detector. Using the same experimental setup, two reference materials (IAEA-RM-375 and IAEA-RM-Soil-6) were measured three times to guarantee the quality of the data. To calculate the background counts, an empty container was also counted under the identical circumstances. In order to determine the specific activities, the net counts were obtained by subtracting the background counts for the identical counting scenario from the counts of each sample. Gamma-ray lines from ^214^Pb (295.221 keV and 351.922 keV) and ^214^Bi (609.320 keV, 1120.310 keV and 1764.551 keV) were used to calculate the radioactive amounts of ^226^Ra. Using the gamma lines of ^208^Tl (583.190 keV and 2614.533 keV) and ^228^Ac (911.205 keV and 968.970 keV), the radioactivity concentrations of ^232^Th were determined. The activity concentrations of ^40^K were determined using the single conversion gamma-ray line 1460.822 keV [28,32–34]. The final radiation concentration in the examined samples was calculated using a weighted mean method.

The minimum detectable activities (MDA) for the radionuclides of interest were calculated by using the following equation [35,36].


$$\begin{array}{c} MDA=\;\frac{\text{2}.\text{7}\text{1}+\text{4}.\text{6}\text{5}\sqrt{N_{B}}}{\varepsilon \times \rho_{\gamma }\times w\times T} \end{array}$$ (1)

where MDA is given in Bq kg^-1^, *N*_*B*_ = net background counts for corresponding photo-peak, ε = counting efficiency corresponding to specific gamma-ray, $\rho_{\gamma }$ = absolute transition probability of the specific gamma-ray, w = mass of the sample in kg, and T = counting time in second. The MDA for the radionuclides of interest was calculated as 0.5 Bq kg^-1^ for ^226^Ra, 0.6 Bq kg^-1^ for ^232^Th and 2.2 Bq kg^-1^ for ^40^K.

The following formula was used to determine each radionuclide’s activity concentration [28,33,36–38]:


$$\begin{array}{c} A_{i}=\;\frac{N}{\varepsilon \times \rho_{\gamma }\times w\times K} \end{array}$$ (2)

where *A*_*i*_ (Bq kg^-1^) is the specific activity, *N* is the net count rate per second (sample minus background), *ε* is the HPGe detector’s efficiency, *ρ*_*γ*_ is the γ-ray emission probability, *w* is the sample’s mass (in kilograms) and K is the product of decay, self-attenuation and cascade summing correction factor.

Equation (3) is the mathematical formula for calculating the uncertainty of the determined radioactivity [28,33,36].


$$\begin{array}{c} Uncertainty=A_{i}\times \;\sqrt{({\frac{u(N)}{N})}^{\text{2}}+({\frac{u(T)}{T})}^{\text{2}}+({\frac{u\left(\rho_{\gamma }\right)}{\rho_{\gamma }})}^{\text{2}}+({\frac{u(w)}{w})}^{\text{2}}+({\frac{u(\varepsilon )}{\varepsilon })}^{\text{2}}} \end{array}$$ (3)

where *T* is the counting time and other symbols have the same meaning as equation (2).

### Radiological hazard parameters

#### Radium equivalent activity.

The radium equivalent activity provides a consistent measure of the total radioactivity released by various radioactive isotopes (in terms of hazardous radium) present in soil, such as ^226^Ra, ^232^Th, and ^40^K. It is possible to calculate the radium equivalent activity using equation (4) [39–42].


$$\begin{array}{c} {Ra}_{eq}=\;M_{Ra}+\text{1}.\text{4}\text{3}M_{Th}+\;\text{0}.\text{0}\text{7}\text{7}M_{K} \end{array}$$ (4)

The mean activity of ^226^Ra, ^232^Th, and ^40^K is represented by $M_{Ra}$, $M_{Th}$, and $M_{K}$, respectively.

#### Hazard index.

To evaluate the radiological risks associated with soil radioactivity, hazard indices are crucial. They provide measurable markers of possible health risks related to radionuclides, including exposure to radiation from the environment and from inside. These factors are essential for developing regulations and policies that aim to reduce health risks while adhering to international radiation safety standards. The external hazard index (*H*_*ex*_) is computed using equation (5) [43,44].


$$\begin{array}{c} H_{ex}=\;\frac{M_{Ra}}{\text{3}\text{7}\text{0}}+\;\frac{M_{Th}}{\text{2}\text{5}\text{9}}+\frac{M_{K}}{\text{4}\text{8}\text{1}\text{0}} \end{array}$$ (5)

The internal hazard index (*H*_*in*_), which evaluates the potential health risk of internal radon exposure and the buildup of its byproducts on lung tissues, is computed by equation (6) [45].


$$\begin{array}{c} H_{in}=\;\frac{M_{Ra}}{\text{1}\text{8}\text{5}}+\;\frac{M_{Th}}{\text{2}\text{5}\text{9}}+\frac{M_{K}}{\text{4}\text{8}\text{1}\text{0}} \end{array}$$ (6)

#### Absorbed dose rate in the air.

Due to the presence of ^226^Ra, ^232^Th, and ^40^K in the studied samples, the absorbed dose rate in the air provides an estimate of the external gamma ray dose received by an individual. At a distance of one meter above the ground, the external absorbed dose rate, *D*, from the gamma rays emitted by the analyzed samples was calculated using equation (7) [30,37,45].


$$\begin{array}{c} D=\;{\text{0}.\text{4}\text{6}\text{2}A}_{Ra}+\;\text{0}.\text{6}\text{0}\text{4}A_{Th}+\;\text{0}.\text{0}\text{4}\text{1}\text{7}A_{K} \end{array}$$ (7)

#### Annual effective dose.

By assessing the outdoor exposures, the yearly outdoor effective doses, *E*_*ff*_, may be obtained. Therefore, the yearly effective dose (mSv/year) was calculated using equation (8) [11,46].


$$\begin{array}{c} E_{ff}=D\times (\;\text{8}\text{7}\text{6}\text{0}\times \text{0}.\text{7}\;\times \text{0}.\text{2})\times {\text{1}\text{0}}^{-\text{6}} \end{array}$$ (8)

According to UNSCEAR 2000 [11], the average yearly effective dose from indoor (0.41) and outdoor (0.07) terrestrial radiation is 0.48 mSv on a worldwide scale. The ICRP [47], IAEA [11], and the Nuclear Safety and Radiation Control Rules-1997 of Bangladesh [48] all suggest an effective dose limit of 1 mSv annually for public exposure under intended exposure conditions.

#### Gamma level index.

A quantitative metric used to evaluate the possible risk of gamma radiation in a particular setting or location is the gamma level index (*I*_*γ*_). The magnitude of gamma radiation is measured by this quantity, which also calculates the potential radiation exposure of nearby people. The gamma level index is obtained using equation (9) [43].


$$\begin{array}{c} I_{\gamma }=\;\frac{M_{Ra}}{\text{1}\text{5}\text{0}}+\;\frac{M_{Th}}{\text{1}\text{0}\text{0}}+\frac{M_{K}}{\text{1}\text{5}\text{0}\text{0}} \end{array}$$ (9)

#### Excess lifetime cancer risk (ELCR).

When assessing the potential health impacts of exposure to carcinogens, one important metric is the excess lifetime cancer risk (ELCR). Together with the pre-existing cancer risk, this measure offers a numerical evaluation of the exposure’s increased cancer risk. To calculate the ELCR, equation (10) is used [49].


$$\begin{array}{c} \text{ELCR}=\;E_{aed}\times A_{lf}\times R_{f} \end{array}$$ (10)

where *E*_*aed*_, *A*_*lf*_, and *R*_*f*_ stand for the equivalent annual effective dose, average lifetime, and the fatal cancer risk factor, respectively. The average lifespan of people in Bangladesh is 72.6 years [50].. For stochastic effects that might impact the general population, the International Commission on Radiological Protection (ICRP) recommends a risk coefficient of 0.05 (Sv^-1^) [47].

### Statistical analysis

Descriptive statistical analysis was performed to summarize the distribution characteristics of the measured radionuclide concentrations in both gas well core samples and surface soil samples. The statistical parameters including mean, median, standard deviation, variance, skewness, kurtosis, minimum, and maximum values were calculated for the activity concentrations of ^226^Ra, ^232^Th, and ^40^K. The mean and median values were used to represent the central tendency of the data, while the standard deviation and variance were used to evaluate the variability of radionuclide concentrations among the samples. In addition, skewness and kurtosis were calculated to assess the distribution pattern and normality of the dataset. Positive skewness indicates right-skewed distributions, whereas negative skewness indicates left-skewed distributions, while kurtosis values provide information on the peakedness or flatness of the distribution compared to a normal distribution. These descriptive statistics provide an overall understanding of the variability and distribution pattern of natural radionuclides in both core and surface samples of FGF.

Principal component analysis (PCA) was applied to the standardized activity concentrations of ^226^Ra, ^232^Th, and ^40^K to investigate the multivariate relationships among the independently measured radionuclides and to identify possible common geological or geochemical controls on their distribution. The derived radiological hazard indices were not included in the PCA because they are deterministic functions of the radionuclide activity concentrations; including them would therefore introduce circularity and would not provide independent statistical information. Prior to PCA, all radionuclide variables were standardized to zero mean and unit variance to remove the influence of different measurement scales, particularly the much higher numerical range of ^40^K compared with ^226^Ra and ^232^Th. The principal components were extracted from the correlation matrix, and the results were evaluated using eigenvalues, percentage variance explained, score plots, loading plots, and biplots.

In addition, the hierarchical cluster analysis (HCA) was performed by using the same parameters as PCA to evaluate the similarity among the samples studied. Ward’s linkage method and Euclidean distance were applied to the standardized data to perform HCA. The combined use of PCA and HCA provides complementary information: PCA identifies the main directions of variance and radionuclide associations, whereas HCA groups samples with similar radiological characteristics. R and RStudio software programs were used to conduct the PCA & HCA. The entire code and the versions of the software used are provided in the Supplementary Materials.

## Results and discussions

### Distribution of NORMs in gas well core (GWC) samples of FGF

The radioactivity levels of eight core samples from well-2 of the FGF of Bangladesh, ranging at approximate depth from 2190 m to 4090 m, are shown in Table 3. The activity concentrations of ^226^Ra in the core samples ranged from 31 ± 3–62 ± 3 Bq kg^-1^, with an average value of 47 Bq kg^-1^. The GWC sample with the lowest radioactivity concentrations of ^226^Ra was found to be deeper (SF-8; 4090 m) than the depth (SF-7; 3770 m) with the highest concentration. Compared to the worldwide average of ^226^Ra (35 Bq kg^-1^: UNSCEAR, 2000 [11]), the average value of ^226^Ra is greater. The mean ^232^Th radioactivity content in the drilling core samples was 75 Bq kg^-1^, with a range of 52 ± 2 Bq kg^-1^ to 93 ± 3 Bq kg^-1^. Here, the core sample (sand sample) with the lowest ^232^Th radioactivity concentration is located at a deeper depth (SF-6; 3625 m) than the depth (SF-4; 3265 m) with the highest ^232^Th radioactivity concentration. According to UNSCEAR (2000) [11], the average value of ^232^Th is more than twice as high as the worldwide average (30 Bq kg^-1^). With an average level of 1806 Bq kg^-1^, the radioactivity concentration of ^40^K in the core samples varied between 1043 ± 47 Bq kg^-1^ to 2219 ± 57 Bq kg^-1^. The core sample with the lowest radioactivity concentration of ^40^K was also found to be deeper (SF-8; 4090 m) than the depth (SF-3; 3260 m) with the highest ^40^K radioactivity concentration. The broad range is probably caused by variations in geological formations. The average value of ^40^K exceeds the worldwide average value of ^40^K by more than four times (400 Bq kg^-1^: UNSCEAR, 2000 [11]). The predominance of potassium-bearing minerals including illite, and K-feldspar within the sandstone–shale sequences of the Sylhet basin is responsible for the comparatively higher activity concentration of ^40^K in the FGF core samples [22]. In comparison to other naturally occurring radionuclides, potassium buildup is further enhanced by diagenetic modification of clay minerals and the feldspathic character of the reservoir sediments [21,25,26].

**Table 3:** The radioactivity concentrations of the NORMs in the gas well core samples from FGF.

Sample ID	^226^Ra (Bq kg^-1^)	^232^Th (Bq kg^-1^)	^40^K (Bq kg^-1^)	Th/Ra
SF-1	58 ± 3	78 ± 3	1767 ± 49	1.3
SF-2	36 ± 3	72 ± 2	1793 ± 47	2.0
SF-3	49 ± 4	90 ± 3	2219 ± 57	1.8
SF-4	57 ± 3	93 ± 3	2137 ± 52	1.6
SF-5	47 ± 3	75 ± 3	1978 ± 50	1.6
SF-6	38 ± 3	52 ± 2	1485 ± 49	1.3
SF-7	62 ± 3	83 ± 3	2029 ± 53	1.3
SF-8	31 ± 3	56 ± 3	1043 ± 47	1.8
**Average**	**47**	**75**	**1806**	**1.6**
Range	31 - 62	52 - 93	1043 - 2219	1.3–2.0
World avg [11].	35	30	400	

With an average value of 1.6, the Th/Ra ratio is comparable across all core samples. Low Th/U ratios are favorable for uranium mineralization; sample SF-7 has the lowest ratio, 1.3, where ^226^Ra activity is at its highest. According to lithological data, all eight of the FGF core samples are shale samples, except for one sand sample (SF-6), which has the lowest radioactive concentration of ^232^Th and relatively low levels of ^226^Ra and ^40^K.

There might be several reasons for the increased radioactivity found in the core samples from the FGF. Firstly, the greater NORM concentrations seen in shale strata are well known. Shale is a sedimentary rock that is rich in organic matter. It frequently contains minerals like clay that can serve as radioactive element hosts. These minerals’ presence in the core samples suggests that the FGF’s geological composition contains a natural source of radiation. Secondly, hydrocarbon reservoirs, which are commonly linked to certain rock types like sandstone and shale, are the FGF’s most well-known feature. In the process of hydrocarbon creation and migration, several processes, including fluid-rock interactions, hydrothermal alteration, and organic matter diagenesis, can concentrate radioactive elements in reservoir rocks. Therefore, the elevated radioactivity levels in the related core samples may be a result of the existence of hydrocarbon reserves inside the FGF. Finally, the concentration and mobility of radioactive elements under the surface can be influenced by environmental variables such as weathering, chemical reactions, and groundwater flow. Radioactive minerals can be transported and leached by groundwater moving through porous and fractured rock formations, which can alter the concentration and distribution of these minerals in core samples. Along with changing the mineralogy of rocks, weathering processes like erosion and dissolution may also release preserved radioactive materials into the environment. In conclusion, complex interactions between geological, hydrological, environmental, and tectonic variables spanning geological time periods are probably the cause of the elevated radioactivity seen in the core samples from the FGF. To accurately assess the radiological dangers associated with the discovery and extraction of hydrocarbons in the region, it is imperative to understand these factors.

### Distribution of NORMs in surface samples of FGF

**Fig 2:**
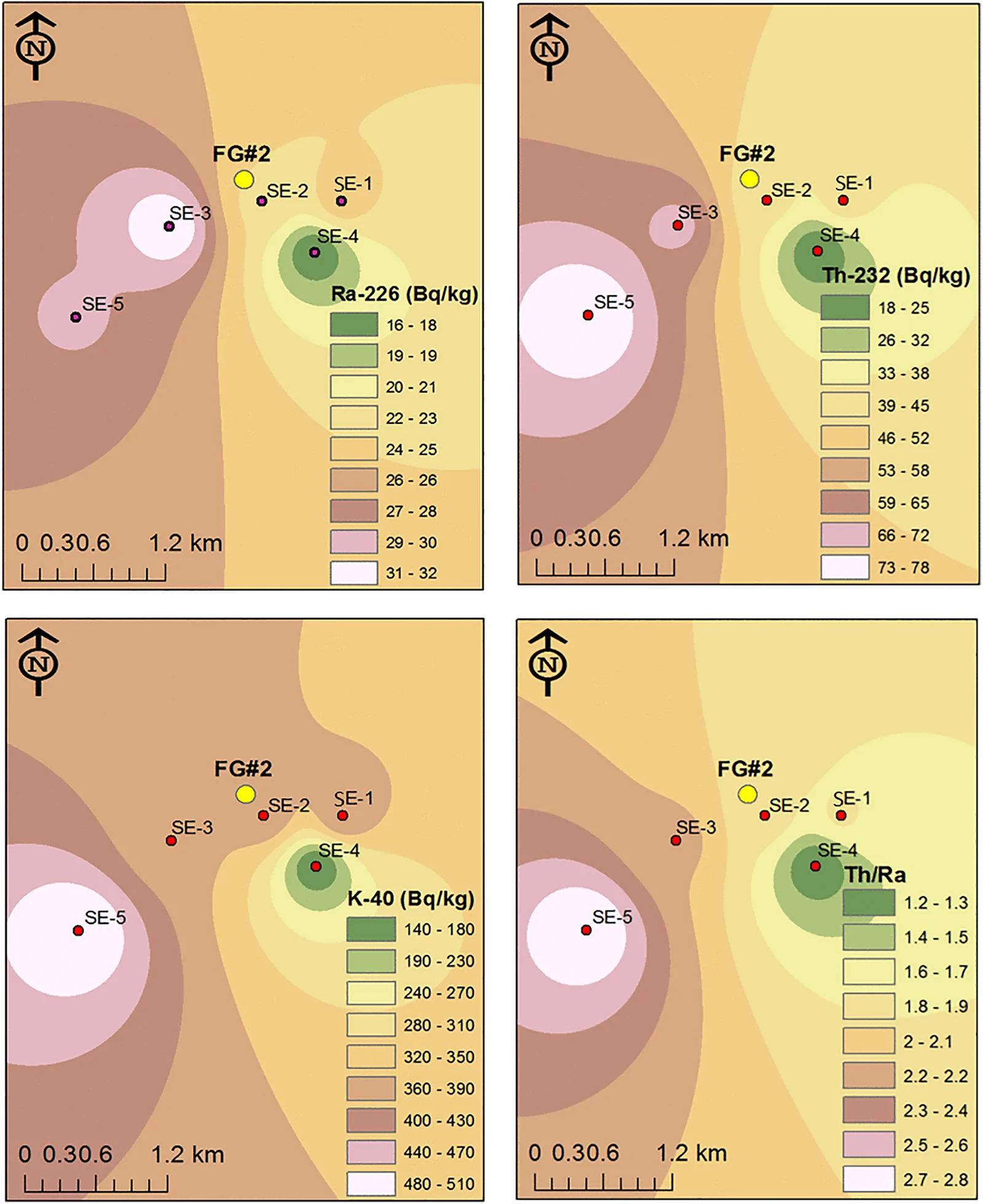
Spatial distribution maps of NORMs in surface samples of Fenchuganj gas field.

In Table 4, the Th/Ra ratio and the specific radioactivity levels of the FGF soil and sediment samples are included together with the corresponding standard deviations (SD). The surface soil samples of the evaporation pond side region of sample SE-4 and SE-3 yielded the lowest and highest radioactivity concentrations of ^226^Ra, respectively. The radioactivity concentrations of ^226^Ra in the investigated area ranged from 16 ± 3–32 ± 4 Bq kg^-1^, with an average value of 24 Bq kg^-1^. Radioactivity concentrations of ^232^Th ranged from 18 ± 2 Bq kg^-1^ to 78 ± 3 Bq kg^-1^, with an average of 49 Bq kg^-1^. The average ^40^K radioactivity level is 361 Bq kg^-1^, with a range of 144 ± 38 Bq kg^-1^ to 516 ± 53 Bq kg^-1^. The identical soil sample from SE-4, which was taken from the southeastern side of the evaporation pond region where chemical wastes are disposed of, likewise contained the lowest radioactive concentrations of ^232^Th and ^40^K. The same soil sample from SE-5, which was taken from beyond the gas field and plant area of FGF, had the highest concentrations of both ^232^Th and ^40^K radioactivity. According to UNSCEAR (2000) [11], the average radioactivity concentrations of ^40^K and ^226^Ra of FGF are lower than the corresponding worldwide average values. The only value that is greater than the equivalent worldwide average, 30 Bq kg^-1^, is the average of ^232^Th [11]. The FGF soil’s higher ^232^Th levels in contrast to their ^226^Ra and ^40^K values are explained by a number of distinct geological, geochemical, and environmental processes. Higher ^232^Th concentrations are a result of the region’s geology, which is rich in thorium-bearing minerals such as feldspars and kaolinite [22]. While potassium and radium are more vulnerable to leaching and groundwater movement, thorium can accumulate due to its resilience to weathering. The removal of ^226^Ra and ^40^K is further aided by soil properties like high permeability and regional climate features like heavy rainfall. Furthermore, ^40^K may be selectively depleted by human activities like farming without having a substantial impact on thorium levels. For better understanding, the spatial distributions of radioactivity concentrations of ^226^Ra, ^232^Th, ^40^K, and Th/Ra in the samples (SE-1 to SE-5) of FGF have been presented in Fig 2.

**Table 4:** The radioactivity concentrations of the NORMs in the surface samples from FGF.

Sample ID	^226^Ra (Bq kg^-1^)	^232^Th (Bq kg^-1^)	^40^K (Bq kg^-1^)	Th/Ra
SE-1	24 ± 3	41 ± 2	391 ± 41	1.7
SE-2	22 ± 4	40 ± 3	387 ± 47	1.8
SE-3	32 ± 4	67 ± 3	366 ± 53	2.1
SE-4	16 ± 3	18 ± 2	144 ± 38	1.2
SE-5	28 ± 4	78 ± 3	516 ± 53	2.8
**Average**	**24**	**49**	**361**	**1.9**
Range	16 - 32	18 - 78	144 - 516	1.2–2.8
World avg [11].	35	30	400	–

The elevated NORMs level in some of the surface samples close to the FGF are also caused by anthropogenic activities, particularly during the rainy season. The overflow of skimming pits and wastewater ponds, among other gas extraction processes, allow ^226^Ra to be incorporated into the soil and spread out across a large region inside and around the gas field’s perimeter. A further factor in the accumulation of NORMs in the surrounding environment is waste byproducts from gas extraction activities, such as sludge and scale. Additionally, radioactive materials can be removed from sludge and deposits by using high-velocity water jets to clean industrial equipment. This allows the materials to be transported through drainage systems to nearby areas, such as skimming pits and waste-dumping evaporation ponds. These regions are particularly vulnerable to flooding during the rainy season, which disperses NORMs at random and raises soil radiation levels.

### Comparison with other studies

The comparison between the NORM concentrations in soil samples from oil and gas production facilities in various nations across the world and those from the current investigation is shown in Table 5. Table 5 shows that, in contrast to Bangladesh, the activity concentration of ^226^Ra is somewhat greater in soil samples taken from Kuwait, Romania, Iraq, China, Nigeria and Turkey, whereas other countries have comparable values. The data for ^232^Th is substantially lower than that of the current research for the other nations. In case of ^40^K, the activity concentration is comparable with other countries. The authors contend that this soil contamination is a result of sludge that contains naturally occurring radionuclides and scale that is removed during facility maintenance or waste disposal procedures [54]. On the other hand, our results are in good agreement with research that was previously carried out at the Shabazpur gas field in Bangladesh except ^40^K, suggesting that the geological conditions in our study region and the field are similar [3]. The geological characteristics of the Shahbazpur and Fenchuganj gas fields are comparable since they are both located in the eastern fold belt of the Bengal Basin. Both areas have Miocene-age sedimentation. They share similar hydrocarbon sources connected to flysch deposits and trap formation tied to tectonic movements. Additionally, they include stacked, multi-layered reservoir sand bodies [63].

**Table 5:** Comparative analysis of NORMs concentrations in samples from oil and gas production sites around the globe.

Country		Activity concentration (Bq kg^-1^)					
		^226^Ra		^232^Th		^40^K	
		Range	Mean	Range	Mean	Range	Mean
Saudi Aribia [51]		8.68 - 156	23.2	–	–	108 - 446	278
Albania [52]		12 - 23	18.3	–	–	326 - 549	413.7
Iraq [53]		18.4 - 97.6	–	11.5 - 42.7	–	176.9 - 485.6	–
Turkey [54]		24.79 - 70.48	46.47		–	–	–
Romania [55]		60 - 330	–	8 - 87	–	53 - 960	–
Ghana [56]		–	–	8.5 - 67.2	26.9	60.4 - 248.9	157
Nigeria [57]		19.2 - 94.2	41	17.7 - 47.5	29.7	107 - 712	412.5
Qatar [58]		–	20.05		16.43		216.69
Kuwait [59]		33.7 - 250.6	85	10.5 - 21.96	13	331 - 449.4	406
Syria [60]		18.9 - 210	–	16.8 - 55.9	–	44 - 213	–
China [61]		16 - 82	57	–	–	–	–
Oman [62]		–	–	5.7 - 9.1	7.1	93 - 293	151
Bangladesh (prior study, other gas field) [3]	Surface soil	26 - 55	40	41 - 82	73	610 - 1203	895
	Core sample	47 - 82	60	75 - 121	94	1481 - 2756	2194
**Bangladesh (this study)**	Surface soil	16–32	**24**	18–78	**49**	144 - 516	**361**
	Core sample	31–62	**47**	52–93	**75**	1043 - 2219	**1806**

### Radiological hazard assessment

**Fig 3:**
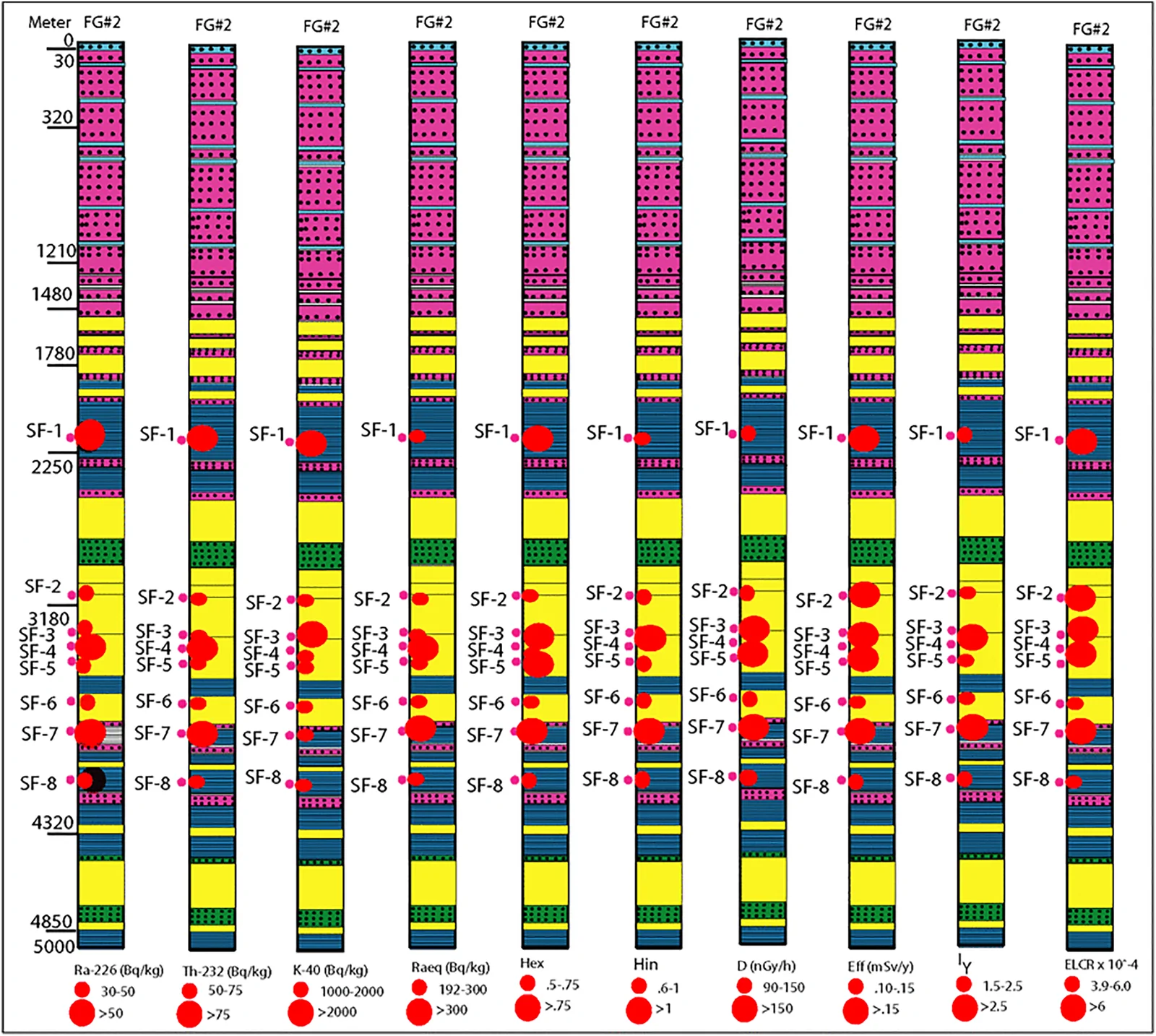
Distribution maps of NORMS and hazard parameters of core samples at various depths of Fenchuganj gas field (well#2).

**Fig 4:**
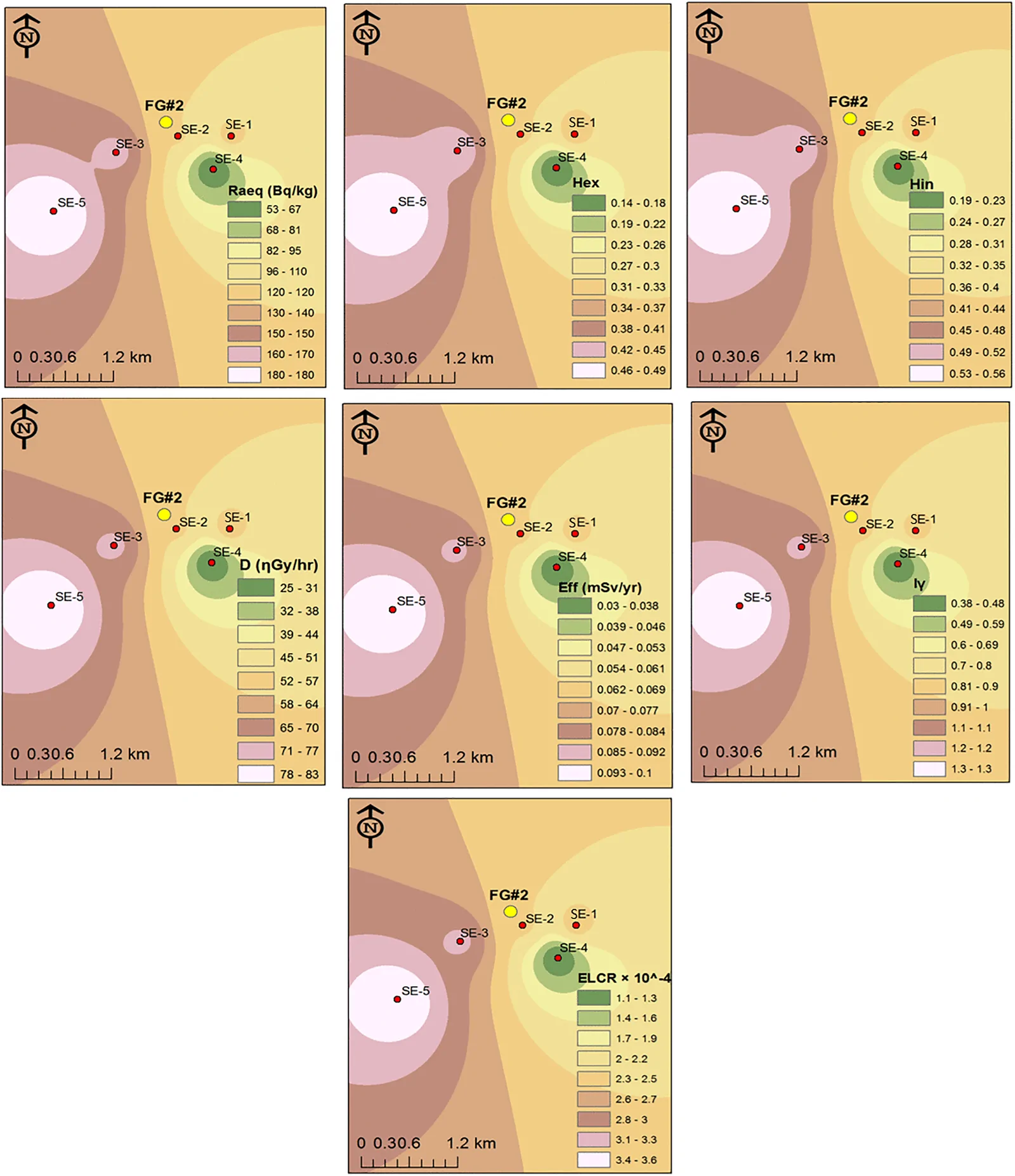
Spatial distributions of radiological hazard indices in soil and sediment samples of Fenchuganj gas field.

The values of the seven radiological hazard indices for the FGF’s core samples are listed in Table 6, while the values for the FGF’s soil and sediment samples are shown in Table 7. Additionally, Fig 3 illustrates the distribution of NORMs (^226^Ra, ^232^Th, and ^40^K) and radiological hazard indices (*Ra*_*eq*_, *H*_*ex*_, *H*_*in*_, *D*, *E*_*ff*_, *I*_*γ*_, and ELCR) in GWC samples of FGF and Fig 4 represents the spatial distributions of radiological hazard indices in the soil and sediment samples of FGF.

**Table 6:** The radiation hazard indices of the core samples from FGF.

Sample ID	*Ra*_*eq*_ (Bq kg^-1^)	*H*_*ex*_	*H*_*in*_	*D* (nGy h^-1^)	*E*_*ff*_ (mSv y^-1^)	*I*_*γ*_	ELCR (×10^−4^)
SF-1	306	0.83	0.98	149	0.18	2.34	6.4
SF-2	277	0.75	0.85	137	0.17	2.16	5.9
SF-3	349	0.94	1.07	172	0.21	2.71	7.4
SF-4	355	0.96	1.11	174	0.21	2.73	7.5
SF-5	307	0.83	0.95	151	0.19	2.38	6.5
SF-6	227	0.61	0.71	112	0.14	1.76	4.8
SF-7	337	0.91	1.08	165	0.2	2.60	7.1
SF-8	191	0.52	0.6	93	0.11	1.46	4.0
**Average**	**294**	**0.79**	**0.92**	**144**	**0.18**	**2.27**	**6.2**
Range	191 - 355	0.52–0.96	0.6–1.11	93 - 174	0.11–0.21	1.46–2.73	4.0–7.5
Recommended values [11]	370	<1	<1	59	0.07	1	2.9

**Table 7:** The radiation hazard indices of the surface samples from FGF.

Sample ID	*Ra*_*eq*_ (Bq kg^-1^)	*H*_*ex*_	*H*_*in*_	*D* (nGy h^-1^)	*E*_*ff*_ (mSv y^-1^)	*I*_*γ*_	ELCR (×10^−4^)
SE-1	113	0.3	0.37	53	0.06	0.83	2.3
SE-2	109	0.29	0.35	51	0.06	0.8	2.2
SE-3	156	0.42	0.51	72	0.09	1.13	3.1
SE-4	53	0.14	0.19	25	0.03	0.38	1.1
SE-5	179	0.48	0.56	83	0.1	1.31	3.6
**Average**	**122**	**0.33**	**0.4**	**57**	**0.07**	**0.89**	**2.5**
Range	53 - 179	0.14–0.48	0.19–0.56	25 - 83	0.03–0.1	0.38–1.31	1.1–3.6
Recommended values [11]	370.0	<1	<1	59	0.07	1	2.9

The Radium equivalent activity (*Ra*_*eq*_) for the GWC samples has shown a variation between 191–355 Bq kg^-1^ with a mean value of 294 Bq kg^-1^, while for soil and sediment samples, it ranged from 53 to 179 Bq kg^-1^ with a mean value of 122 Bq kg^-1^. These values are lower than the recommended value of 370 Bq kg^-1^ [11].

Table 6 shows that the average value of *H*_*ex*_ for GWC samples of FGF is 0.79, with a range of 0.52 to 0.96. The *H*_*in*_ had a mean value of 0.92 and ranged from 0.6 to 1.11. Thus, for *H*_*ex*_ and *H*_*in*_, the GWC sample values are near about and around one, respectively (Fig 3). Additionally, the average *H*_*ex*_ value for the soil and sediment samples is 0.33, with a range of 0.14 to 0.48, and the average *H*_*in*_ value is 0.4, with a range of 0.19 to 0.56 (Table 7). Therefore, all FGF soil samples are radiologically risk-free in terms of *H*_*ex*_ and *H*_*in*_; nevertheless, it is recommended that samples be regularly inspected since soil and sediment samples may be contaminated by NORMs originating from gas-abstraction activities.

All the GWC samples have absorbed dose rates (*D*) that are around two to three times more than the suggested threshold of 59 nGy h^-1^ [11]. It varied between 93 and 174 nGy h^-1^, with an average of 144 nGy h^-1^, as seen in Table 6 and Fig 3. The *D*-value ranges from 25 to 83 nGy h^-1^, with an average of 57 nGy h^-1^ for the surface samples (soil and sediment) (Table 7 and Fig 4). However, samples SE-3 and SE-5 had absorbed dose rates that were higher than the advised threshold. Drilling chemical waste was discovered to have been deposited at these sites. However, the average yearly effective dose rate for GWC samples is 0.18 mSv y^-1^, which is higher than the worldwide average for outdoor soil effective dose. The average for surface samples was 0.07 mSv y^-1^, which is consistent with the recommended value [11].

The gamma representative level index (*I*_*γ*_) values of every GWC sample are higher than the corresponding recommended value of 1.0, as shown in Table 6. Compared to the surface samples (soil and sediment), the values of most GWC samples are much higher and more than twice as high as the equivalent recommended value (Table 7). For GWC samples, the average *I*_*γ*_-value is 2.27, with a range of 1.46 to 2.73. The surface samples had an average *I*_*γ*_-value of 0.89, ranging from 0.38 to 1.31.

With the exception of two GWC samples of SF-6 and SF-8, all the FGF GWC samples had ELCR values that are more than twice as high as the equivalent recommended threshold of 2.9 × 10^−4^ (Table 6). Two (SE-3 and SE-5) surface samples had ELCR values that are higher than the associated suggested value (Table 7). The ELCR level index values for GWC samples in this case vary from 4 × 10^−4^ to 7.5 × 10^−4^, with an average of 6.2 × 10^−4^. With a range of 1.1 × 10^−4^ to 3.6 × 10^−4^, the surface samples’ average ELCR value is 2.5 × 10^−4^. Therefore, if appropriate safeguards are not implemented, working at this gas exploration site poses a long-term health risk.

### Statistical analysis results

#### Descriptive statistics.

**Fig 5:**
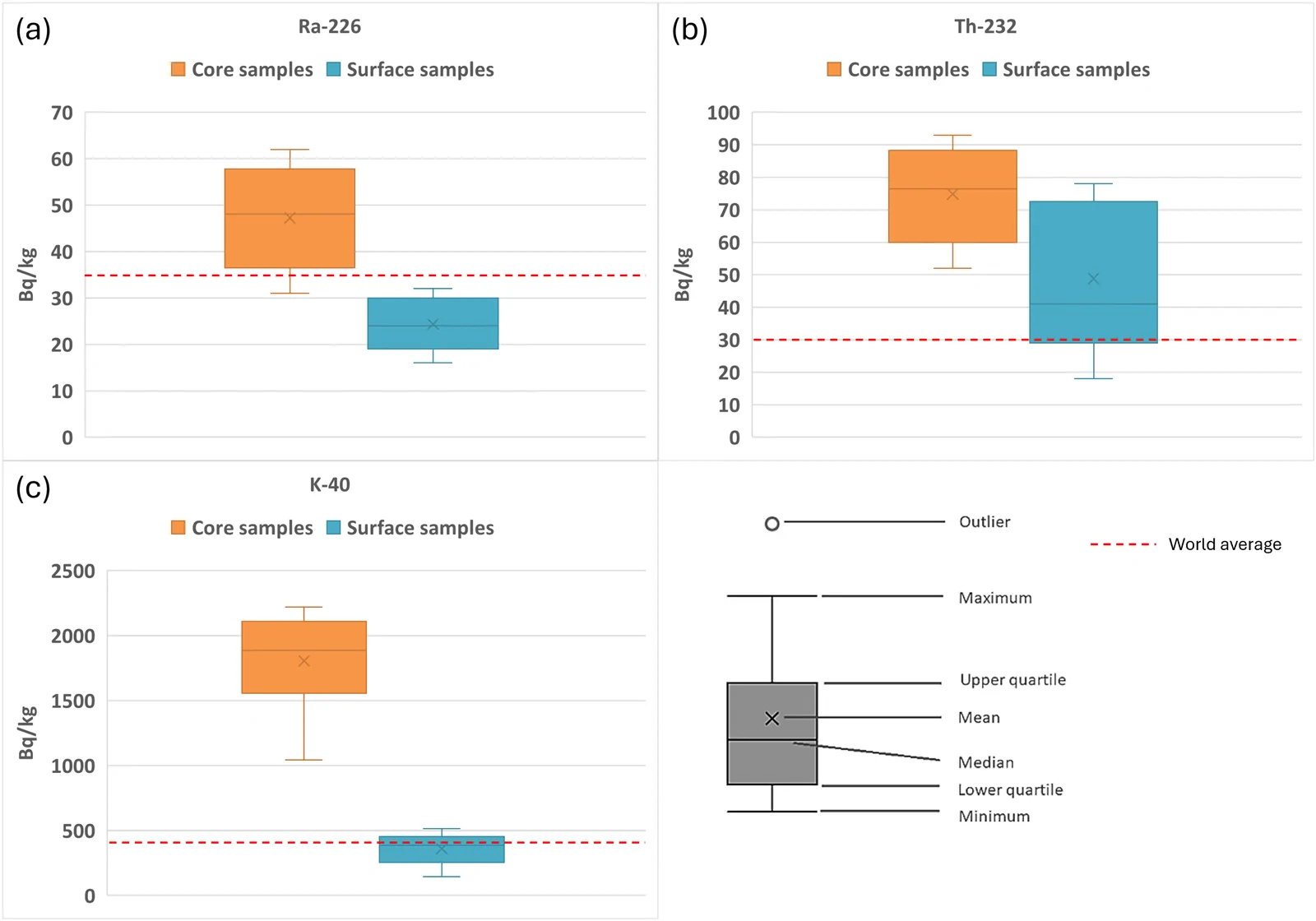
Box and whisker plot for radioactivity concentrations of the NORMs in the GWC and surface samples from FGF.

The descriptive statistical parameters of the activity concentrations of ^226^Ra, ^232^Th, and ^40^K in gas well core samples and surface soil samples of FGF are presented in Table 8. For both the GWC and surface samples, the close agreement between the mean and median values indicates that the radionuclide concentrations are relatively symmetrically distributed within the dataset. The observed ranges reflect moderate variability among the samples. The radionuclide concentrations in surface samples were considerably lower than those observed in the GWC samples, suggesting a higher accumulation of NORMs in the subsurface geological formations. For easier understanding, a comparison between the NORMs concentrations of GWC samples and surface samples are also depicted in Fig 5 by box and whisker plots.

**Table 8:** Descriptive statistics of NORMs in the studied samples from FGF.

Sample	Parameter name	^226^Ra	^232^Th	^40^K
Gas well core samples	Mean	47.25	74.88	1806.38
	Median	48	76.5	1885.5
	Standard deviation	11.39	14.74	386.62
	Variance	129.64	217.27	149477.4
	Kurtosis	−1.58	−0.79	1.15
	Skewness	−0.14	−0.52	−1.17
	Minimum	31	52	1043
	Maximum	62	93	2219
Surface samples	Mean	24.4	48.8	360.8
	Median	24	41	387
	Standard deviation	6.07	23.83	134.83
	Variance	36.8	567.7	18178.7
	Kurtosis	−0.14	−1.23	2.56
	Skewness	−0.23	0.02	−1.09
	Minimum	16	18	144
	Maximum	32	78	516

The standard deviations for the core samples were 11.39, 14.74, and 386.62 Bq kg − ¹ for ^226^Ra, ^232^Th, and ^40^K, respectively, indicating noticeable variability, particularly for potassium. In surface samples, the corresponding standard deviations were 6.07, 23.83, and 134.83 Bq kg − ¹, suggesting moderate spatial variability in radionuclide concentrations across the study area.

For the GWC samples, skewness values were −0.14 for ^226^Ra, −0.52 for ^232^Th, and −1.17 for ^40^K, indicating slightly negatively skewed distributions, particularly for potassium. For the surface samples, skewness values were −0.23 for ^226^Ra, 0.02 for ^232^Th, and −1.09 for ^40^K. The skewness value close to zero for ^232^Th indicates a nearly symmetric distribution, while ^40^K shows a moderately negative skewness, suggesting the presence of several relatively high potassium concentrations within the dataset.

In the GWC samples, kurtosis values were −1.58 for ^226^Ra and −0.79 for ^232^Th, indicating platykurtic distributions, which are flatter than a normal distribution. In contrast, ^40^K exhibited a positive kurtosis value (1.15), indicating a leptokurtic distribution, meaning the presence of heavier tails and occasional extreme values. For the surface samples, ^226^Ra (−0.14) and ^232^Th (−1.23) also exhibited platykurtic distributions, whereas ^40^K showed a high positive kurtosis value (2.56), suggesting a more peaked distribution with a higher probability of extreme values. These statistical characteristics indicate that potassium exhibits greater variability compared with radium and thorium in both sample types.

#### Principal component analysis.

**Fig 6:**
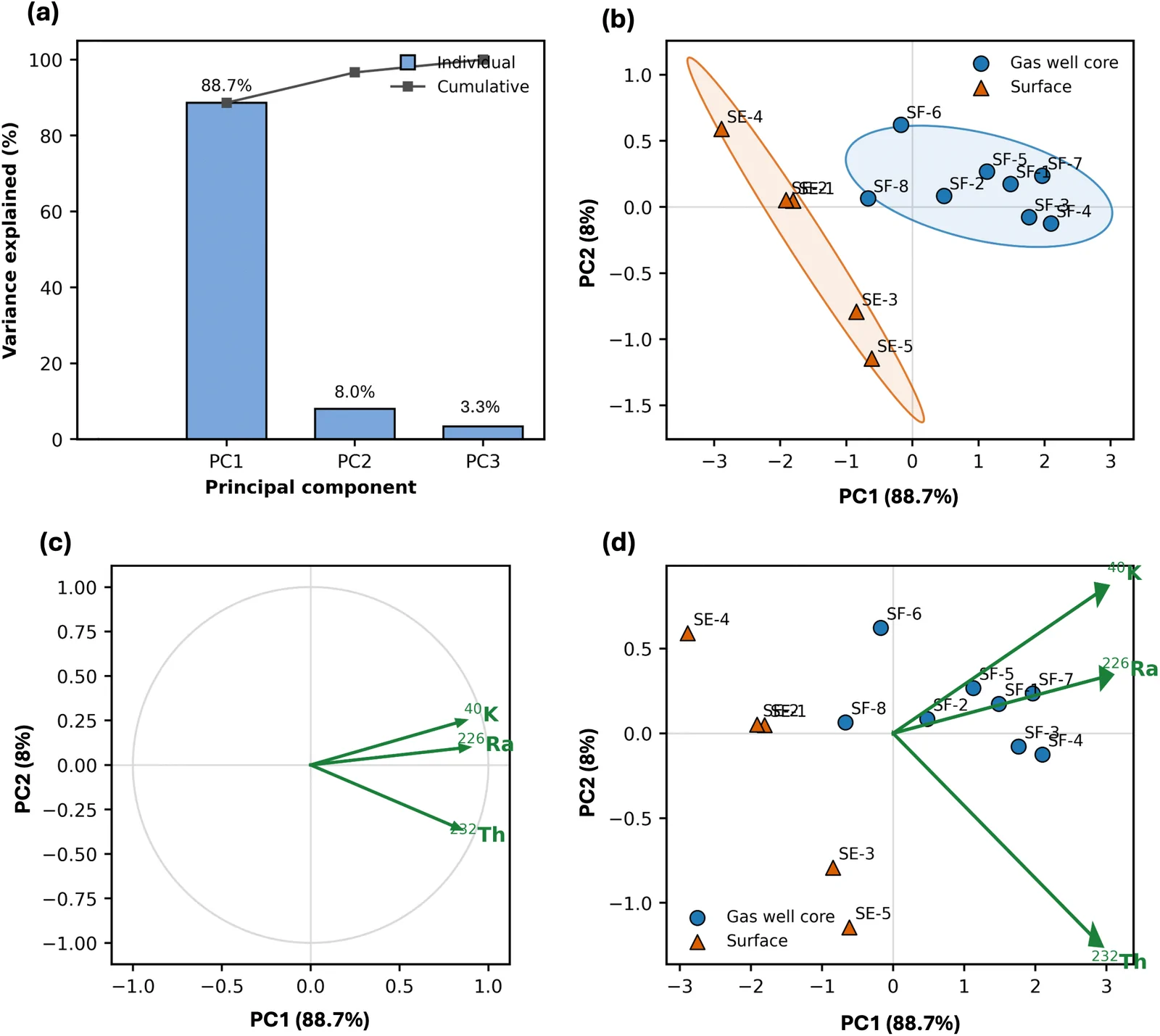
Principal component analysis (PCA) plots; (a) scree plot, (b) score plot, (c) loading plot, and (d) biplot.

Principal component analysis was performed using only the measured activity concentrations of ^226^Ra, ^232^Th, and ^40^K. The extracted principal components and their explained variances are shown in Table 9 and Fig 6(a). The first principal component (PC1) explained 88.7% of the total variance, while the second principal component (PC2) explained 8.0%. Together, PC1 and PC2 accounted for 96.7% of the total variance in the radionuclide dataset, indicating that the three measured radionuclides can be effectively represented by the first two principal components. The third component explained only 3.3% of the variance and therefore contributed little additional information.

**Table 9:** Eigenvalues and variance explained by each principal component.

Principal component	Eigenvalue	Variance explained (%)	Cumulative variance (%)
PC1	2.659	88.7	88.7
PC2	0.239	8.0	96.7
PC3	0.101	3.3	100.0

The PCA score plot (Fig 6(b)) clearly separates most gas well core samples from the surface samples along PC1. The gas well core samples plot mostly on the positive side of PC1, consistent with their elevated activities of ^226^Ra, ^232^Th, and ^40^K compared with the surface samples. In contrast, most surface samples occur on the negative side of PC1, reflecting lower radionuclide concentrations and comparatively lower natural radioactivity. This separation confirms that the subsurface core materials and surface samples have distinct multivariate radionuclide signatures.

The PCA loading plot (Fig 6 (c)) shows that ^226^Ra, ^232^Th, and ^40^K all have positive loadings on PC1 (^226^Ra = 0.963, ^232^Th = 0.918, and ^40^K = 0.943). This indicates that PC1 represents a common radionuclide enrichment component. Samples with high positive PC1 scores are characterized by relatively high activities of all three radionuclides, whereas samples with negative PC1 scores correspond to lower radionuclide activities. Therefore, PC1 is interpreted as reflecting the overall natural radioactivity level of the samples, mainly controlled by lithological and mineralogical characteristics of the FGF.

PC2 represents a secondary source of variability. The loading structure indicates that PC2 mainly differentiates the relative behavior of ^232^Th from those of ^226^Ra and ^40^K. This component may reflect minor lithological or geochemical variations, including differences in clay mineral abundance, potassium-bearing minerals, and the relative distribution of uranium/radium- and thorium-bearing phases. The position of individual samples on PC2 suggests that, although the overall radioactivity level is mainly described by PC1, some samples have slightly different radionuclide proportions.

The PCA biplot (Fig 6(d)) further supports the interpretation that the higher radioactivity of the core samples is associated with simultaneous enrichment of ^226^Ra, ^232^Th, and ^40^K. The relatively close orientation of the loading vectors indicates a positive association among the radionuclides, suggesting that their distribution is largely controlled by common geological factors. This is consistent with the occurrence of shale-dominated subsurface formations and thorium-bearing minerals such as feldspars and kaolinite in the sandstone-shale sequences of the Bengal basin [22].

#### Hierarchical clustering analysis.

**Fig 7:**
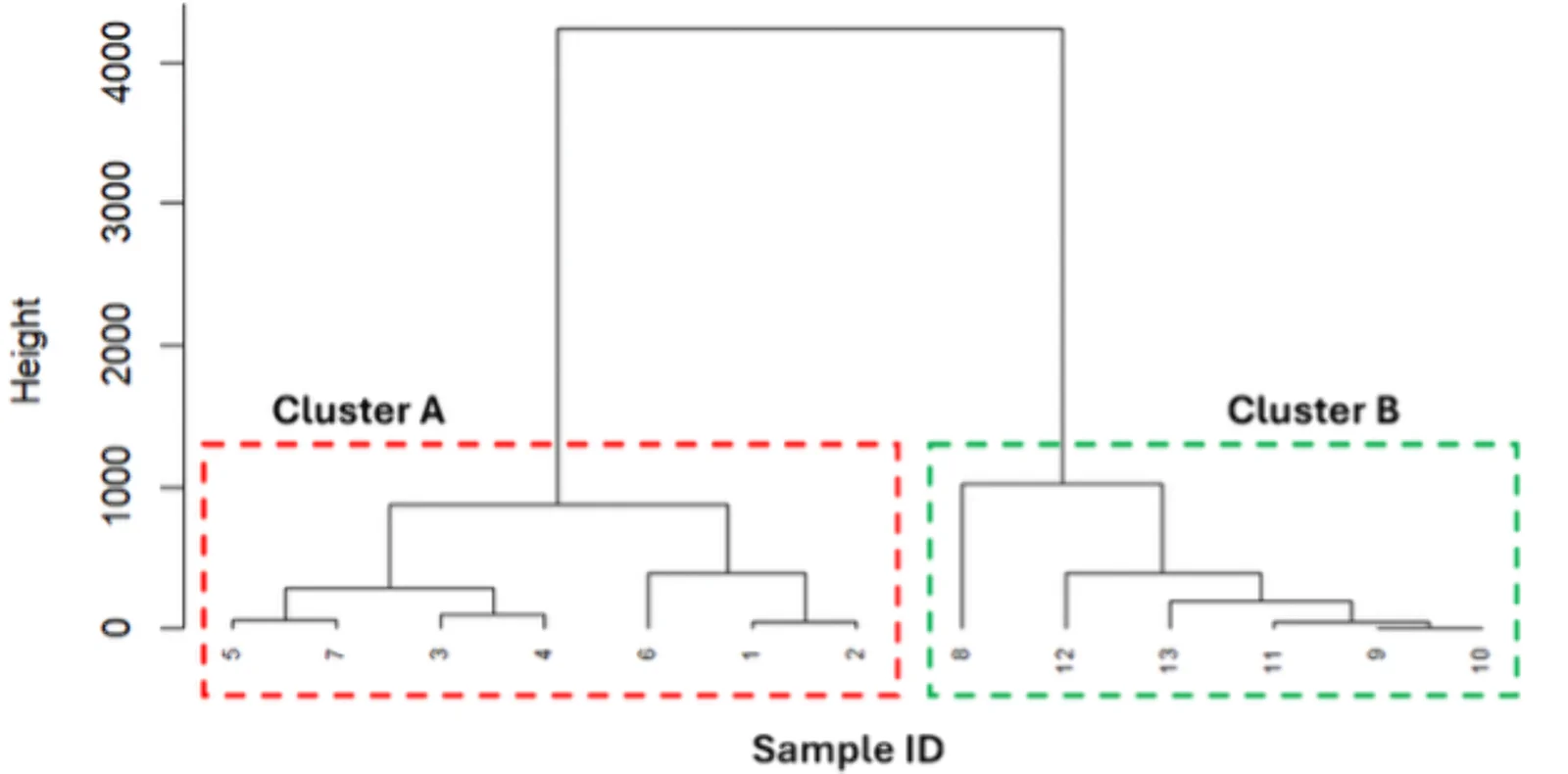
Dendrogram showing the Hierarchical clustering analysis (HCA) results.

In Fig 7, GWC samples of FGF are represented by 1–8, while surface samples are denoted by 9–13. The HCA dendrogram (Fig 7) provides complementary evidence for the PCA results. The HCA dendrogram reveals the formation of two distinct clusters, cluster A and cluster B, indicating variations in radiological characteristics among the samples. A significant distance between cluster A and cluster B further supports their different origins.

Samples within the same cluster are likely to share similar environmental or lithological features. It is evident that the samples taken from the FGF gas well cores make up cluster A. Therefore, the mineral composition, lithological formation, geological origin, or environmental processes controlled their features. Apart from sample 8, which is a core sample, cluster B is made up of the samples taken from the FGF’s surface. The properties of these samples were mostly impacted by environmental factors (rainfall, groundwater movement, erosion, weathering, etc.) and anthropogenic activities (industrialization, mining, farming, etc.). The agreement between PCA and HCA confirms that the distinction between core and surface samples is not based on a single radionuclide alone but on their combined multivariate radionuclide characteristics.

### Management guidelines

Exposure to radiation from contaminated soil can cause several health issues, such as an increased risk of developing cancer and other diseases brought on by radiation. Long-term contact with polluted soil can also harm wildlife, ecosystems, and perhaps taint water supplies. Given the threats that the gas well core samples from the FGF pose to the environment and worker’s health, it is imperative that a few suggestions be put into practice in order to successfully reduce these risks. The following management guidelines are recommended to ensure safe handling of core materials and to minimize potential occupational exposure.

(i) Regular radiological monitoring should be implemented during drilling, core extraction, and maintenance operations. Periodic measurement of radionuclide activity concentrations and ambient gamma dose rates can help identify areas or materials with elevated natural radioactivity. Monitoring programs should follow internationally recognized radiation protection recommendations provided by the UNSCEAR and the IAEA.
(ii) Since elevated radionuclide concentrations were observed in subsurface core samples, appropriate radiation protection practices should be adopted for workers involved in drilling and core handling activities. These measures may include: (a) use of personal protective equipment (PPE) such as gloves, masks, and protective clothing; (b) limiting the duration of exposure to core materials with higher radioactivity; (c) maintaining adequate distance and shielding where feasible during handling and storage. These practices can significantly reduce potential external and internal radiation exposure.
(iii) Handling and processing of core samples may generate dust particles containing NORMs. To reduce the risk of inhalation exposure, core samples should be handled in well-ventilated areas. Dust suppression techniques should be applied during drilling and sample preparation. Workers should use respiratory protection where dust generation is significant. These measures will help minimize internal exposure through inhalation pathways.
(iv) Core samples with elevated radioactivity should be stored in designated and properly labelled storage areas to prevent unnecessary exposure. Proper containment and controlled access to storage facilities can further enhance safety during long-term storage.
(v) Although the surrounding surface soils show radionuclide concentrations within typical background levels, periodic environmental monitoring is recommended to ensure that operational activities do not lead to long-term accumulation of radionuclides in the environment.
(vi) Personnel involved in drilling and sample handling should receive basic radiation safety training, including awareness of NORM, safe handling procedures, and appropriate protective measures.
(vii) All operational and monitoring activities should comply with national radiation safety regulations and international safety guidelines established by the IAEA. Adherence to these standards will ensure that occupational and environmental exposures remain within acceptable limits.

Overall, the implementation of these management practices will help reduce potential occupational exposure associated with subsurface geological materials while maintaining safe operational and environmental conditions in gas field activities.

## Conclusion

This study investigated the activity concentrations of natural radionuclides and associated radiological hazard parameters in gas well core samples and surface samples of the Fenchuganj gas field. The results indicate that the activity concentrations of radionuclides (^226^Ra, ^232^Th, and ^40^K) in the gas well core samples are generally higher than the corresponding global average values reported by the UNSCEAR. Consequently, the calculated radiological hazard indices, including absorbed gamma dose rate, annual effective dose, gamma level index, and ELCR, are comparatively elevated for the subsurface core materials. These findings suggest that although the radionuclide enrichment is primarily associated with the geological formations at depth, occupational exposure during drilling, core handling, and maintenance activities may require appropriate radiological monitoring and safety considerations. The observed radiological characteristics therefore highlight the importance of implementing standard radiation protection practices in operational environments involving subsurface geological materials. In contrast, the surface soil samples show radionuclide concentrations largely within the typical natural background range, with only a slight increase in the activity concentration of ^232^Th compared with global reference values. However, the corresponding radiological hazard parameters for surface soils remain within internationally accepted safety limits, indicating minimal radiological risk for the general environment and public exposure. Overall, the study provides important baseline information on the distribution of NORMs in the Fenchuganj gas field, contributing to a better understanding of radiological characteristics, occupational exposure considerations, and environmental safety in gas field regions.

## Supporting information

S1 File RStudio code for PCA and HCA. (TXT)
